# Gender influence on heart rate variability in Cameroonian handballers

**DOI:** 10.1016/j.jsampl.2026.100145

**Published:** 2026-06-15

**Authors:** Franck Yvan Deugoue Djientcheu, Marcel Azabji-Kenfack, Danielle Claude Bilanda, Madeleine Chantal Ngoungoure, Rodrigue Fifen Ngapout, Arnaud Galvani Nekam, Loick Nguegang Mbolang, Aicha El Ramadan Nsangou Malane, Ulrich Membe Femoe, Mireille Poumeni Kameni, Paul Désiré Djomeni Dzeufiet

**Affiliations:** a Laboratory of Animal Physiology, Faculty of Sciences, University of Yaoundé I, Yaoundé, Cameroon; b Laboratory of Physiology, Faculty of Medicine, University of Yaoundé I, Yaoundé, Cameroon; c Faculty of Sciences, University of Bamenda, Bamenda, Cameroon

**Keywords:** Sport, Sinus variability, Sub-Saharan Africa, Women

## Abstract

**Background:**

Heart rate variability (HRV) analysis is a useful tool for evaluating the heart's ability to adjust to external loads. This study aimed to assess the gender impact on cardiac autonomic modulation response in Cameroonian handballers.

**Method:**

ology: A prospective analytical study was conducted on sports teams. Participants were divided into three groups; people who practiced little or no sporting activity (sedentary, male), those who were regularly physically active in a handball team (male and female athletes). Resting Heart Rate (HR) was recorded for 10 min and analysed.

**Results:**

Male players had significantly higher HR than women (68.75 vs 60.38; p < 0.001). SDNN (Standard Deviation of the inter-beat interval of normal sinus beats), RMSSD (Root Mean Square of the Successive Differences) and pNN_50_ (proportion of successive NN intervals that differ by more than 50 ms) were lower in sedentary people (16.22; 9.97; 0.16) than in male handballers group (29.00; 16.44; 2.15; p < 0.001 and p < 0.05). Very-low-frequency relative power was higher in male players than in female players (30.82 vs 21.29; p < 0.05). However, male Low-Frequency relative power, High-Frequency absolute and relative power (69.26; 3.46 and 6.96) were significantly lower than in female players (77.12; 4.19 and 9.67, with p < 0.05; p < 0.05; p < 0.01 respectively). The effect size for gender was low for the Low–Frequency parameter.

**Conclusion:**

The findings revealed an association between sports practice and high HRV values but do not allow for a clear conclusion regarding the impact of gender on the parasympathetic and sympathetic parameters determining HRV.

## 1 Introduction

Heart rate variability (HRV) denotes fluctuations in the intervals between heartbeats, or instantaneous heart rate in diverse circumstances [1,2]. As a physiological phenomenon, it extends beyond the heart rate (HR) measurement to provide insights into the sinoatrial node's response to inputs from the parasympathetic and sympathetic nervous systems [3,4]. It is a non-invasive method for assessing the autonomic nervous system activity at the cardiac level, reflecting the regulation of autonomic balance, vascular tone, and blood pressure [2,5,6]. It can be influenced by numerous factors such as physical activity [5,7]. Nevertheless, the HRV use as a practical monitoring instrument for allostatic load has been infrequent. It could, however, be a simple and potentially useful tool for diagnosing overreaching athletes and monitoring their responses to training [8,9]. A correlation has been identified between an elevated HRV and enhanced fitness, greater responsiveness to aerobic training, elevated levels of physical activity, and diminished work-related stress [2,8,10]. In addition, HRV is a tool for assessing athletes' recovery capacity [11].

Numerous factors can influence HRV. These include recording conditions, data processing, and subject-related variables [[12], [13], [14], [15], [16], [17]]. While the contribution of some of these factors to HRV is well established, others remain controversial. For instance, gender – one of the subject-related variables – demonstrates significant disparities in its impact on HRV. Studies have reported an increase, a decrease, or no difference in HRV indices between men and women [[17], [18], [19]]. To better understand the impact of gender on HRV parameters, this study was conducted to analyse the effects of sports practice and gender on HRV.

## 2 Materials and methods

### 2.1 Participants and study design

A prospective, analytical study was conducted on several sports teams in the city of Yaoundé in the Centre region of Cameroon. Participants were either active handball players (men and women) or non-athletic individuals (sedentary, male) aged at least 18 years old who had provided their consent. The participants' age, anthropometric (weight, height), and hemodynamic (HR [with POLAR H10®, *Polar Electro Inc., Singapore*] and blood pressure [with OMRON® *Automatic Upper Arm Blood Pressure Monitor*, M3 {HEM-7154-E}]) parameters were recorded. Their Body Mass Index (BMI) and mean blood pressure were also calculated. This study (ethical clearance N°1587 CE/CRERSHC/2020) was conducted with strict respect for the integrity of the participants, in compliance with the relevant regulations and complying with the COVID-19 pandemic control barrier measures. Participants were informed that they could withdraw from the study at any time, without any consequence.

### 2.2 HRV analysis

The HR was recorded and processed as described by Djientcheu et al. [20]. The parameters of interest calculated using the *Kubios HRV Standard*® software were represented by the time domains [the Standard Deviation of the NN interval (SDNN), the square Root of the Mean of the Sum of the Squares of Differences between adjacent NN intervals (RMSSD), the proportion derived by dividing the number of interval differences of successive NN intervals greater than 50 ms by the total number of NN intervals (pNN50)], frequency analysis (High Frequency-HF; Low Frequency-LF; Very Low Frequency-VLF, LF/HF ratio and Total power-TP) and non-linear analysis (Standard Deviations of the scattergram-SD_1&2_ and SD_2_/SD_1_ ratio). These HRV parameters were then exported and stored in *Microsoft Excel* to form our database [20]. These indices were subsequently analyzed according to whether they exclusively reflect SNP activity (RMSSD, NN_50_& pNN_50_, HF, SD_1_) or not [3,12,15,21,22].

### 2.3 Statistical analysis

The data were analysed using the statistical software packages *SPSS* 23.0 (IBM SPSS® Inc, Chicago, Illinois, USA) and *GraphPad Prism* 8.0 (GraphPad Software, San Diego, California, USA). The means ± mean standard error (MSE) comparison were conducted using a one-way ANOVA and Tukey's multiple comparison post-test. A one-way ANCOVA was performed to assess the gender impact on specific HRV parameters; this was achieved through the Levene test or its non-parametric equivalent, followed by the Bonferroni post-test. A significance threshold of p < 0.05 was employed.

## 3 Results and discussion

### 3.1 Results

#### 3.1.1 Study population

**Fig. 1:**
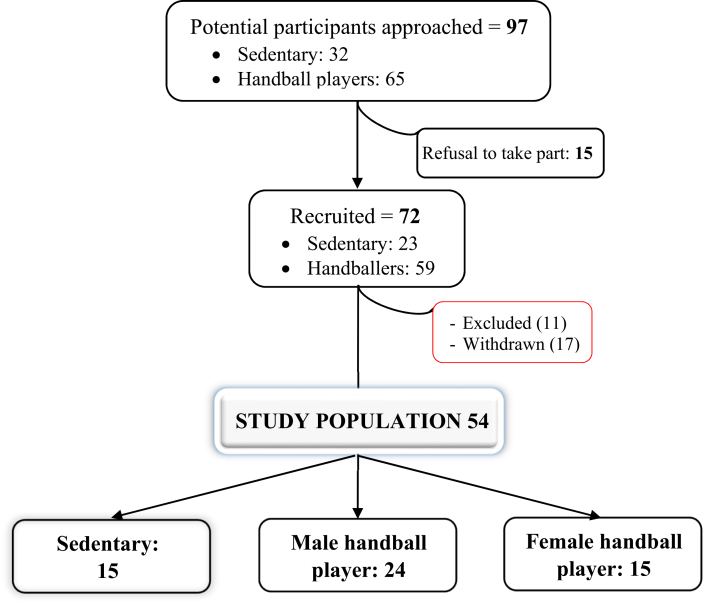
Study flow chart.

Of the 54 people selected to participate in the study, 72.2% (39/54) were handball players [44.4% male players (24/54) and 27.8% female players (15/54)]. The remaininCg 27.8% (15/54) constituted the sedentary control group (Fig. 1).

#### 3.1.2 Clinical and sporting characteristics of the study population

##### 3.1.2.1 Clinical characteristics

The mean blood pressure of male handballers was statistically higher than that of female players (p < 0.05). The BMI of female handball players was approximately 7.0% higher than that of male players, and we found no significant difference in the age of the participants (Table 1).

**Table 1:** Repartition of the participants according to their clinical data.

Variables	Sedentary (n = 15)	Handball Player	
		Male (n = 24)	Female (n = 15)
Age (years)	26.27 ± 1.25	29.46 ± 1.05	29.27 ± 1.07
BMI (kg/m^2^)	26.38 ± 0.94	25.20 ± 0.75	26.96 ± 1.13
DBP (mm Hg)	69.27 ± 1.93	76.25 ± 2.04	71.20 ± 1.76
SBP (mm Hg)	124.90 ± 3.08	122.80 ± 2.58	111.70 ± 2.74 **^α^**
MBP (mm Hg)	87.80 ± 1.70	91.76 ± 2.06	84.71 ± 1.75 **^α^**

Each value represents the mean ± MSE, ^c^ p < 0.001 significant difference from sedentary people, ^α^ p < 0.05 significant difference from male players. **BMI**, body mass index; **DBP**, diastolic blood pressure; **MBP**, mean blood pressure; **SBP**, systolic blood pressure.

##### 3.1.2.2 Sporting practice

**Fig. 2:**
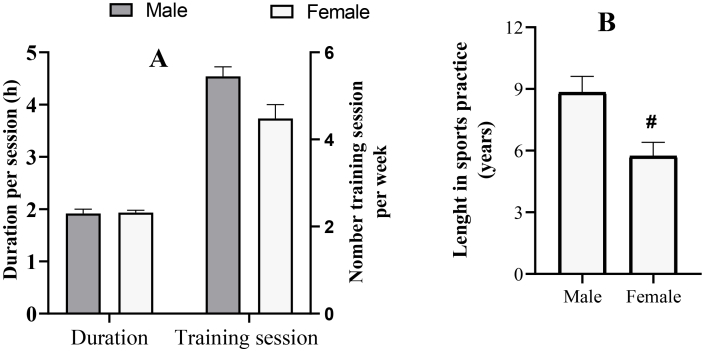
Repartition of players according to their respective sporting activities; **A:** duration per session and number of weekly training sessions; **B:** number of years of sports practice. Each bar represents the mean ± MSE, ^#^p < 0.05 significant difference from male players.

In terms of sporting activities, male handballers had significantly more years of sporting experience (p < 0.05) (Fig. 2B), with an average number of weekly training sessions slightly superior to those of women handball players (Fig. 2A).

#### 3.1.3 Sinus variability characteristics

##### 3.1.3.1 Analysis of the time domain

**Fig. 3:**
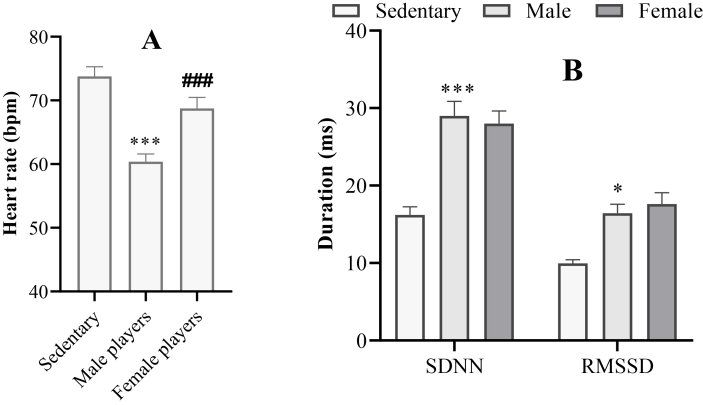
Participants distribution in accordance with the time domain parameters; **A:** heart rate at rest; **B:** SDNN and RMSSD. Each bar represents the mean ± MSE, ∗p < 0.05 ∗∗∗p < 0.001 significant difference from sedentary people; **^###^**p < 0.001 significant difference from male players. **HR:** Heart rate; **RMSSD:** Root Mean Square of the Successive Differences; **SDNN:** The standard deviation of the inter-beat interval of normal sinus beats.

The HR of female handballers at rest was significantly higher (p < 0.001) than that of male players (Fig. 3A). The SDNN, RMSSD, and pNN_50_ of sedentary people were significantly lower (p < 0.001, p < 0.05, and p < 0.05 respectively) than those of male handballers. However, until no statistical difference was observed between the sexes for these parameters, pNN_50_ was round 37% higher in male handballers than in women (Fig. 3B, Table 2).

**Table 2:** Population repartition in accordance with the time domain analysis.

Parameters	Sedentary n = 15	Handball Player	
		Male (n = 24)	Female (n = 15)
RR (ms)	840.00 ± 8.83	994.50 ± 24.91 **^c^**	855.20 ± 25.03 **^γ^**
Min HR (bpm)	68.30 ± 1.32	54.73 ± 1.28 **^c^**	62.57 ± 1.39 **^β^**
Max HR (bpm)	77.77 ± 1.10	74.26 ± 1.91	83.67 ± 1.79 **^β^**
NN_50_ (beats)	0.87 ± 0.34	5.33 ± 1.01	4.73 ± 0.86
pNN_50_ (%)	0.16 ± 0.06	2.15 ± 0.38 **^a^**	1.57 ± 0.29

Each value represents the mean ± MSE, ^a^ p < 0.05 ^c^ p < 0.001 significant difference from sedentary; **^β^** p < 0.01 **^γ^**p < 0.001 significant difference from male players. **Min & Max HR:** Minimum & maximum registered heart rate at rest; **NN50:** The number of pairs of successive NN (R–R) intervals that differ by more than 50 ms; **pNN50:** The proportion of NN50 divided by the total number of NN (R–R) intervals; **RR:** duration between two consecutive R waves.

##### 3.1.3.2 Frequency analysis

###### 3.1.3.2.1 Analysis of parasympathetic parameters (HF, SD_1_)

The HF absolute power (log) was significantly lower in sedentary people than in male handball players (p < 0.01) for which the HF absolute and relative power were statistically lower than those of female players (p < 0.05; p < 0.01). SD1 in the sedentary participants were lower (p < 0.001) compared to that of male handballers, and this indice was similar between male and female handball players (Table 3).

**Table 3:** Distribution of the study population in accordance with the frequency analysis parameters.

Variables	Sedentary n = 15	Handball Player	
		Male (n = 24)	Female (n = 15)
**VLF**			
Absolut power (ms^2^)	45.27 ± 2.90	200.70 ± 29.53	182.30 ± 39.33
Absolut power (log)	3.66 ± 0.08	4.86 ± 0.16 **^c^**	4.63 ± 0.22
Relative power (%)	16.21 ± 0.64	30.82 ± 2.67 **^c^**	21.29 ± 2.60 **^α^**
**LF**			
Absolut power (ms^2^)	170.80 ± 16.31	658.30 ± 83.09 **^c^**	618.30 ± 58.14
Absolut power (log)	5.04 ± 0.15	6.03 ± 0.19 **^a^**	6.21 ± 0.23
Relative power (%)	75.35 ± 0.86	69.26 ± 2.63	77.12 ± 1.75 **^α^**
Normalise power (n.u.)	87.23 ± 1.47	89.18 ± 1.24	84.06 ± 2.02
**HF**			
Absolut power (ms^2^)	22.95 ± 1.33	42.76 ± 5.15	65.11 ± 6.14
Absolut power (log)	2.50 ± 0.16	3.46 ± 0.16 **^b^**	4.19 ± 0.15 **^α^**
Relative power (%)	8.39 ± 0.38	6.96 ± 0.42	9.67 ± 0.68 **^β^**
Normalise power (n.u.)	12.79 ± 1.18	10.90 ± 0.76	11.09 ± 0.82
**Normalized power**			
Total power (ms^2^)	246.40 ± 18.04	927.30 ± 94.12 **^c^**	864.90 ± 87.61
Ratio LF/HF	7.55 ± 0.58	9.07 ± 0.60	8.23 ± 0.70
**Non-linear analysis**			
SD_1_ (ms)	5.89 ± 0.34	11.44 ± 0.84 **^b^**	11.29 ± 0.88
SD_2_ (ms)	20.14 ± 0.83	36.32 ± 2.40 **^c^**	35.00 ± 1.51
Ratio SD_2_/SD_1_	3.50 ± 0.14	3.31 ± 0.19	3.31 ± 0.25

Each value represents the mean ± MSE, **^a^** p < 0.05 **^b^** p < 0.01 **^c^** p < 0.001 significant difference from sedentary people, **^α^** p < 0.05 **^β^** p < 0.01 significant difference from male players. **HF**, High frequency; **LF**, Low frequency. **SD1 & 2**, standard deviations of the scattergram; **VLF**, Very low frequency.

###### 3.1.3.2.2 Analysis of other HRV parameters

VLF (absolute and relative power), LF (absolute power), TP and SD_2_ indice of sedentary participants were significantly lower than those of male handball players (p < 0.001 and p < 0.05). Additionally, the relative power of VLF and LF bands was significantly (p < 0.05) greater and lower respectively, in male than in female handball players. There was no statistical difference in TP, LF/HF ratio and SD_2_/SD_1_ ratio according to gender (Table 3).

#### 3.1.4 Covariance analysis

After controlling for years of sports practice and resting HR, there was a significant difference in LF normalized power [F (1, 35) = 7.263, p = 0.011], HF absolute power [F (1, 35) = 14.904, p < 0.001] and HF relative power [F (1, 35) = 10.109, p = 0.003] concerning the gender. Post hoc showed more significant differences in LF normalized power and HF absolute power between male and female players (p = 0.011 and p < 0.001 in contrast to p > 0.05 and p > 0.01 initially).

In addition, the effect size for gender was low for these parameters. The partial Eta squared value shows that 17.2%, 29.9%, and 22.4% of the variance in LF, HF absolute, and relative power are explained by gender when years of sports practice and baseline HR are controlled (Table 4).

**Table 4:** Handballers distribution in accordance with the ANCOVA analysis.

Variables	Adjusted mean		F	df	η^2^ value	p-value
	Male	Female				
VLF (%)	29.07 ± 2.55	24.10 ± 3.41	1.120	1–35	0.031	0.297
LF (%)	69.66 ± 2.51	76.49 ± 3.35	2.189	1–35	0.059	0.148
LF (n.u.)	90.00 ± 1.46	82.74 ± 1.95 **^a^**	7.263	1–35	0.172	0.011
HF (log)	3.31 ± 0.16	4.43 ± 0.21 **^c^**	14.904	1–35	0.299	0.000
HF (%)	6.82 ± 0.53	9.91 ± 0.71 **^b^**	10.109	1–35	0.224	0.003

Each value represents the mean ± MSE, ^a^ p < 0.05 ^b^ p < 0.01 ^c^ p < 0.001 significant difference from male players. **df:** degree of freedom; **HF:** High Frequency; **LF**, Low Frequency; **η^2^**, Partial Eta squared value; V**LF**, Very Low Frequency.

### 3.2 Discussion

The objective of this study was to compare cardiac autonomic modulation responses in Cameroonian according to gender and sporting practice. The SDNN, RMSSD, pNN_50_, SD_1,_ and SD_2_ values of sedentary participants were significantly lower (p < 0.001, p < 0.05 and p < 0.01) than those observed in the male handball players group. We found that female handball players had significantly higher mean resting HR, HF, and LF (p < 0.001; p < 0.01; p < 0.05) than male handball players. On the other hand, they had significantly lower VLF (p < 0.05) as well as lower pNN50 value compared to their male peers. Furthermore, the effect size for gender was low for LF normalized power, HF absolute, and relative power.

The resting HR of sedentary participants was significantly greater than that of male handball players. Similar results are reported in the literature. This could be due to a hyperactivation of the parasympathetic system and/or a training-related increase in plasma volume related to regular sports practice, allowing for a greater degree of expansion of the heart [7,20,23]. The fact that the female handball players' HR at rest was higher than that of male players could lead to a reduction in the variability of their HR as a result of the shorter inter-beat interval [24].

There is an established relationship between physical activity and favourable modifications in body weight and blood pressure. In this study, female players had significantly lower systolic and mean blood pressure than their male peers. Gender differences in autonomic regulation reflect greater parasympathetic activity in women, which is suggestive of supporting a cardioprotective mechanism in women [25]. These findings could thus be attributed to a greater reduction in cardiac output and a lesser increase in total peripheral resistance in comparison with that observed in the male players [24].

No significant difference was observed in BMI between the various groups. These results diverge from those previously reported by Ngongang et al. [26] and Brunkhorst and Kielstein [27], who worked respectively with athletes playing in different sports (handball, football, etc.) and with volunteers of both sexes and two sports (cyclists and triathletes). The observed outcomes may be attributed to the fact that the athletes of this study had virtually a similar weekly training load, regardless of their gender. However, the higher BMI of female players compared with male players could be a factor limiting their performance [28,29].

It has been established that athletes with greater sports experience are more likely to perform better [30,31]. This would be the case for the male handball players in our study, who have accumulated the greatest length of time engaged in sporting activities and undertake the highest volume of weekly training compared to the female handballers. However, this remains hypothetical because, in addition to training time, performance also depends on the quality of training, participation in competitions, and factors such as BMI [30,32].

The low HRV parameter values obtained in this study, particularly those from the temporal analysis (SDNN and RMSSD), when compared to those documented in the literature, could be a consequence of a short recovery time and/or reflect a state of fatigue [33]. This is because participants had a recovery period of less than 24 h after their previous training session before participating in this study. Indeed, as Thorpe et al. [7] have demonstrated, HRV can be reduced in the days following intense exercise as a result of sympathetic dominance. This low variance in R–R intervals is frequently linked to diminished baroreceptor sensitivity and indicates a potential modification in the regulation of cardiac vagal tone [34].

The SDNN and RMSSD values were significantly lower in sedentary participants than those observed in male handballers. This could be attributed to the disparity in physical fitness levels between athletes and non-athletes of this study [20]. Indeed, elevated HRV values indicate a greater degree of parasympathetic activation relative to sympathetic activation in an athlete. This suggests therefore a superior capability to respond to subsequent challenges [2]. Furthermore, the fact that SD_1_ and SD_2_ are significantly higher in male handball players compared to sedentary people would be indicative of improvements in HRV in the short and long term [35].

In this study, female players had significantly higher HR and HF than male players. On the other hand, they had significantly lower LF, as well as a relatively lower pNN_50_ value. High HR values associated with lower pNN_50_ values, like those observed in female handball players, may indicate predominant sympathetic control of heart rate in these athletes, who would then exhibit greater sympathetic tone at rest [36]. The finding that the female handball players in our study were overweight, in contrast to the male handball players, could be a potential explanation for these outcomes [5,36]. Indeed, Almeida-Santos et al. found lower pNN_50_ values in female handball players compared to male handball players when analysing 24-h recordings. Consequently, these researchers concluded that overall autonomic regulation is weaker in male subjects and individuals with higher BMI [16].

Similar findings have been documented by other researchers. In their meta-analysis, Koenig and Thayer [17] found that women had lower SDNN, VLF, and LF power values, but higher HF power values compared to men. Moreover, Umetani et al. [21] reported that before the age of 30, as in the present case, women had lower HRV measurements than their male peers. These outcomes indicate a reduction in overall HRV, which may be attributed to an enhancement in sympathetic activity and/or a diminution in parasympathetic activity among female handball players [2,5,8]. This could be attributed to low testosterone levels in female players or alternatively, the consequence of cyclical hormonal variations inherent to the female physiology [12,37]. In contrast, Vesterinen et al. [18] observed no gender-related differences in the adaptation to sport over time. Differences among the participants with regard to eumenorrhea and hormonal contraceptive utilisation may be the underlying reason for the absence of analogous observations [19].

Nonetheless, a difficulty in interpreting our data arises from disparities in the HRV parameters of male and female handball players, particularly those assessing parasympathetic activity (especially HF and pNN_50_). Such contradictions in our findings make it difficult to establish a general pattern of HRV behaviour according to gender. In fact, the interactions between the PNS and the SNS are complex, nonlinear and often non-reciprocal; moreover, confounding due to respiratory mechanics and resting HR creates ambiguity concerning the contributions of the PNS and the SNS to the different parameters of HRV during the measurement period [12]. Conversely, heterogeneity in training response represents a wider phenomenon encompassing other clinically significant factors, such as blood pressure, HR fluctuations in response to exercise and recuperation, or glucose and lipid metabolism [38,39]. Further data collection and an enlarged sample size could facilitate the clarification of potential gender differences through analysis.

## 4 Conclusion

The findings of this study suggest higher values of HRV parameters according to sport practice. However, the presence of contradictions in the parameters that reflect parasympathetic activity in female handball players complicates the determination of a definitive position regarding the influence of gender on HRV. Nevertheless, assessments on larger population sizes, or even with long-term recordings, would likely help to address this issue. Moreover, further research is required among team-sport players over extended training periods, utilising laboratory-based measures of physiological responses (e.g., neuroendocrine and inflammatory markers) to enhance comprehension of HRV responses to training and their associations with gender.

## Work limitations

The primary constraints of this study were the failure of participants to adhere fully to the prescribed condition, including the requirement for complete rest and the prohibition of interaction with others. This could have affected the precision of the data collected. We were also powerless to control the environmental conditions in the experimental room. Moreover, we encountered difficulties in the collection of precise data regarding the menstrual cycle and the utilisation of hormonal contraceptives among the female handball players.

## Data availability

The data that support our findings are accessible from the corresponding author upon reasonable request.

## Funding

This work was not supported by any external funding sources beyond those affiliated with the research team.

## Declaration of competing interest

The authors declare that they have no known competing financial interests or personal relationships that could have appeared to influence the work reported in this paper.
